# Radiological Changes After Craniovertebral Junction Stabilization: Evaluation of Established Measurements and Novel Cranial and Caudal Anterior Atlantodental Interval Parameters

**DOI:** 10.3390/jcm15187111

**Published:** 2026-09-13

**Authors:** Nurullah Kösmene, Efecan Erişken, İsmail Ertan Sevin, Hasan Kamil Sucu

**Affiliations:** 1 Yalova Training and Research Hospital, Yalova 77200, Türkiye; 2 Department of Neurosurgery, Faculty of Medicine, İzmir Katip Çelebi University, İzmir 35620, Türkiye; efecaneriskenn@gmail.com (E.E.); seviner67@yahoo.com (İ.E.S.); hksucu@gmail.com (H.K.S.)

**Keywords:** craniovertebral junction, craniovertebral stabilization surgery, kyphosis, measurement methods

## Abstract

**Background/Objectives:** Craniovertebral junction (CVJ) stabilization alters complex anatomical relationships that may not be fully characterized by a single radiological parameter. This study evaluated the responsiveness of established CVJ measurements after stabilization and explored the observer reliability and postoperative behavior of separate cranial and caudal anterior atlantodental interval (AADI) measurements. **Methods:** This retrospective radiological case series included 26 patients who underwent CVJ stabilization between 2000 and 2022. Eleven parameters were measured on preoperative and immediate postoperative mid-sagittal computed tomography (CT) reconstructions by two independent observers, each completing two measurement sessions, with each observer blinded to the other observer’s measurements and to his own previous session. Reliability was evaluated using intraclass correlation coefficients (ICCs), and paired comparisons were adjusted using the Benjamini–Hochberg procedure. **Results:** Postoperative reductions were observed in odontoid distances from the Chamberlain (q = 0.019), McGregor (q = 0.040), and McRae lines (q = 0.019). Cranial AADI decreased from 5.66 ± 3.31 to 4.17 ± 2.44 mm (q = 0.009), and caudal AADI decreased from 4.81 ± 2.98 to 3.57 ± 2.32 mm (q = 0.019). However, the within-patient cranial–caudal difference score did not change significantly after stabilization (mean change, −0.25 mm; 95% CI, −0.96 to 0.47; *p* = 0.472; Cohen’s dz = −0.19). Intraobserver ICCs ranged from 0.880 to 0.987, while single-measure interobserver ICCs ranged from 0.784 to 0.899. **Conclusions:** Established craniometric distances and separate cranial and caudal AADI measurements demonstrated postoperative radiological changes with good-to-excellent observer reliability. In this cohort, segmented AADI and angular parameters showed different patterns of postoperative change; however, their relative responsiveness was not directly compared. Segmented AADI should currently be regarded as an exploratory radiological parameter, as its diagnostic thresholds, clinical validity, and association with patient outcomes have not yet been established.

## 1. Introduction

The craniovertebral junction (CVJ) refers to the complex anatomical and biomechanical structure connecting the skull base and the upper cervical vertebrae. Stability of this region ensures balanced head and neck movements while protecting the medulla spinalis and brainstem [1,2]. Disruption of its anatomical integrity can lead to severe neurological deficits, respiratory problems, and even life-threatening complications [2].

CVJ pathologies may arise from congenital anomalies (e.g., platybasia, basilar invagination), traumatic injuries (e.g., odontoid fractures, atlanto-occipital dislocation), inflammatory diseases (e.g., rheumatoid arthritis, spondyloarthropathies), or neoplastic processes. While traumatic lesions often require urgent intervention, congenital anomalies typically follow a progressive course and, if not diagnosed early, may cause irreversible neurological damage [2,3].

Surgical treatment of CVJ pathology has evolved considerably over time. Early approaches primarily focused on bony decompression, including procedures such as transoral odontoidectomy and foramen magnum decompression [4]. However, stabilization surgeries have become the mainstay of treatment over time. The advent of screw–rod systems has significantly improved biomechanical stability while reducing morbidity and mortality rates [5]. Today, lateral mass screws, C1-C2 screw–rod systems, and occipitocervical plate systems are widely used [5].

The clinical diagnosis of craniovertebral instability relies heavily on radiological findings. Numerous measurement parameters have been proposed for diagnosis and follow-up [6,7]. However, due to the complex anatomy and biomechanical dynamics of the CVJ, no consensus exists on an “ideal” measurement method, and new approaches continue to emerge [7]. Theoretically, preoperative measurement values should change significantly after stabilization. Despite the importance of this topic, to our knowledge, no previous study has directly compared pre- and postoperative craniovertebral measurements.

Craniovertebral kyphosis is increasingly recognized as an important component of deformity, particularly in patients with basilar invagination and atlantoaxial dislocation. Previous studies have demonstrated that surgical reduction and stabilization can improve craniovertebral alignment, generally assessed using parameters such as the clivo-axial angle, C0-C2 angle, and conventional atlantodental interval [6,8,9]. However, several established measurements require distant cranial landmarks, including the posterior hard palate, clivus, basion, and opisthion, or require technically demanding angular constructions. These landmarks may be incompletely visualized or difficult to identify in patients with complex congenital anatomy or postoperative metallic artifacts [10,11].

The atlantoaxial complex permits coupled angulation and translation, while its stability is predominantly maintained by the transverse and alar ligaments [12,13]. Conventional anterior atlantodental interval (AADI) represents the C1–dens relationship using a single distance; however, it may not reflect local divergence between the opposing surfaces of the anterior C1 arch and the odontoid process [14]. Anatomically, a kyphotic or divergent C1–dens configuration may produce a greater separation cranially than caudally. However, whether separate cranial and caudal AADI measurements provide additional information about local sagittal alignment before and after stabilization has not been investigated previously. We therefore hypothesized that separate cranial and caudal AADI measurements could characterize this local sagittal relationship and exhibit measurable postoperative changes following stabilization.

Accordingly, this preliminary retrospective radiological case series aimed to evaluate: (1) the preoperative to postoperative responsiveness of established craniovertebral measurements, (2) the responsiveness and observer reliability of segmented cranial and caudal AADI measurements, and (3) the exploratory behavior of the C1 anterior arch-odontoid angle following stabilization.

## 2. Materials and Methods

### 2.1. Patient Selection

This retrospective study was approved by the Ethics Committee of İzmir Katip Çelebi University (protocol code 0050; approval date: 15 February 2024). A total of 26 patients who underwent CVJ stabilization at İzmir Atatürk Training and Research Hospital between November 2000 and December 2022 were retrospectively analyzed.

All cases in which any cranial bone segment was surgically fixed to any cervical vertebra were included. In cases in which the lateral masses of C1 were fused with the occipital condyles, fusion between C1 and any subaxial cervical vertebra was also considered craniovertebral stabilization and included in the study. Patients with a history of previous CVJ stabilization who underwent revision surgery due to failure were excluded. Demographic characteristics, etiology, comorbidities, and surgical details were recorded.

### 2.2. Surgical Technique

All patients underwent stabilization through a posterior approach. In the early years, a combination of wire and graft was used; however, over the past 15 years, screw–rod systems have become the standard technique. The cervical vertebral levels used as fixation points, the type of instrumentation employed (plate, rod, wire, screw, etc.), the specific anatomical structures utilized for fixation on the vertebra (lateral mass, pedicle, lamina, pars interarticularis, transfacet, etc.), and the cranial fixation technique (midline or bilateral, using screws, rods, wires, etc.) were evaluated.

### 2.3. Measurement Methods

CT examinations were obtained preoperatively and postoperatively on day 1 to evaluate craniovertebral junction alignment and the adequacy of surgical stabilization. For the purposes of this study, CT examinations obtained on postoperative day 1 were defined as immediate postoperative CT examinations. Images were reviewed and radiological measurements were performed using the web-based DICOM viewer integrated into Probel PACS (Probel Yazılım ve Bilişim Sistemleri A.Ş., İzmir, Türkiye). All CT examinations were acquired with the patient in the supine, head-first position. Neutral head and neck alignment without intentional flexion or extension was intended for all examinations as part of the routine clinical imaging protocol. However, because the images were obtained for clinical purposes and analyzed retrospectively, no dedicated positioning device or quantitative positional criterion was used to confirm identical head and neck orientation between the preoperative and postoperative examinations. Images were reconstructed using a bone algorithm with a slice thickness of 1–2.5 mm. All radiological measurements were performed on mid-sagittal multiplanar CT reconstructions when the relevant anatomical landmarks were clearly identifiable. Images with substantial artifacts, incomplete visualization, or inadequate reconstruction quality were considered unsuitable for the corresponding measurement. Analyses were conducted using the available measurements for each radiological parameter. For each parameter, preoperative–postoperative comparisons were restricted to patients in whom the relevant anatomical landmarks were assessable on both examinations. Thus, the sample size for each paired analysis represented the number of patients with complete preoperative and postoperative measurements for that specific parameter.

Radiological measurements were independently performed by two observers with experience in craniovertebral junction imaging. All measurements were performed on preoperative and postoperative CT images using a predefined radiological measurement protocol. The observers were blinded to each other’s measurements, and each observer independently performed all measurements twice, with the two measurement sessions conducted at least 2 weeks apart to minimize recall bias.

**Figure 1:**
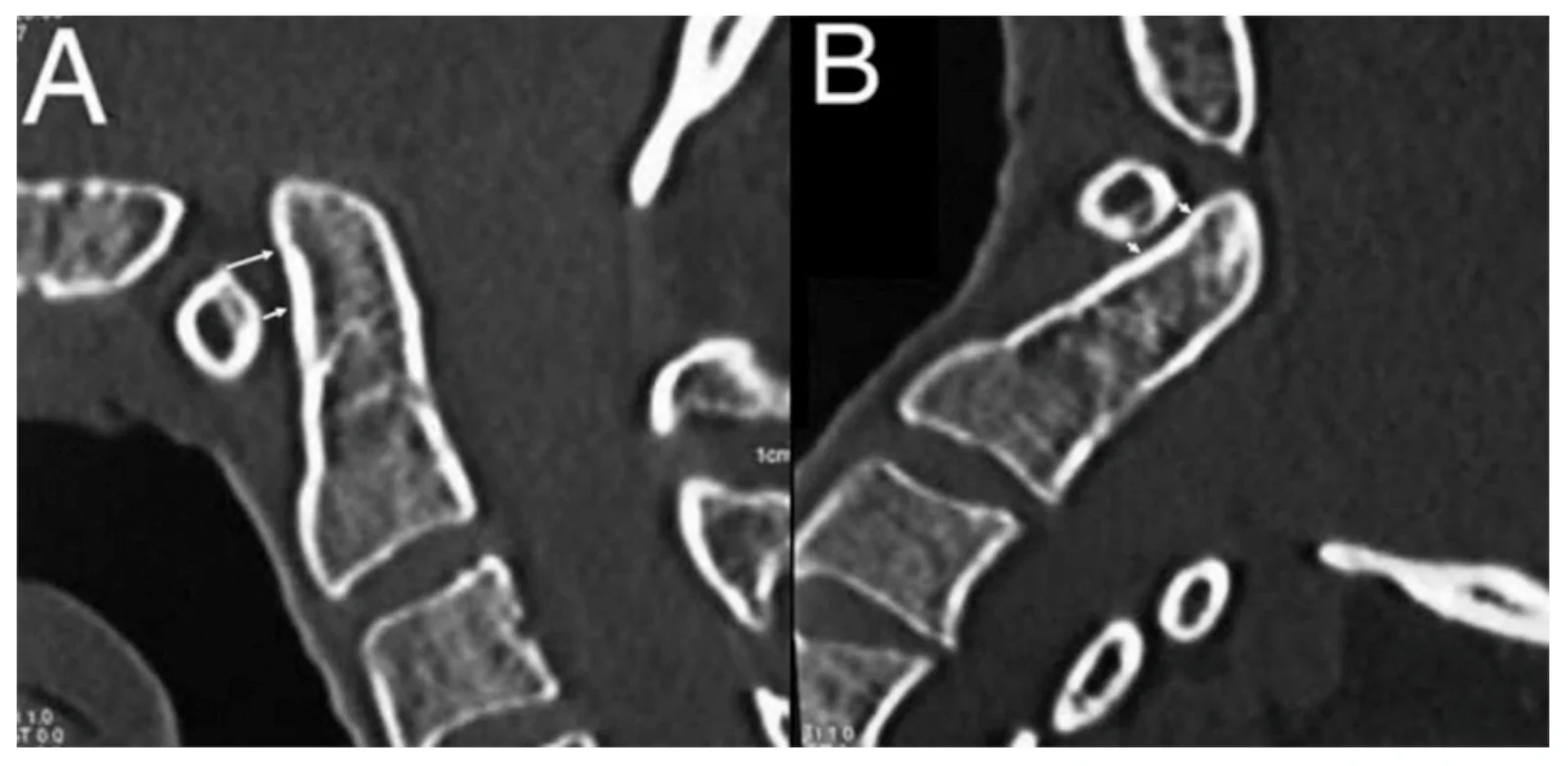
Measurement of cranial and caudal AADI. (**A**) Pre-op; (**B**) post-op.

Classical measurements (Chamberlain, McGregor, McRae), angular measurements (clivus canal, clivodens, atlantoaxial, C0-C1, C0-C2), and new parameters (separate cranial and caudal AADI, and the C1 anterior arch-odontoid angle) were measured according to a predefined standardized measurement protocol. The cranial AADI was measured on mid-sagittal CT reconstructions as a perpendicular line drawn from the dorsocranial corner of the anterior arch of the atlas to the anterior surface of the odontoid process (Figure 1). The caudal AADI was measured on the same mid-sagittal section as a perpendicular line drawn from the dorsocaudal corner of the anterior arch of the atlas to the anterior surface of the odontoid process (Figure 1).

**Figure 2:**
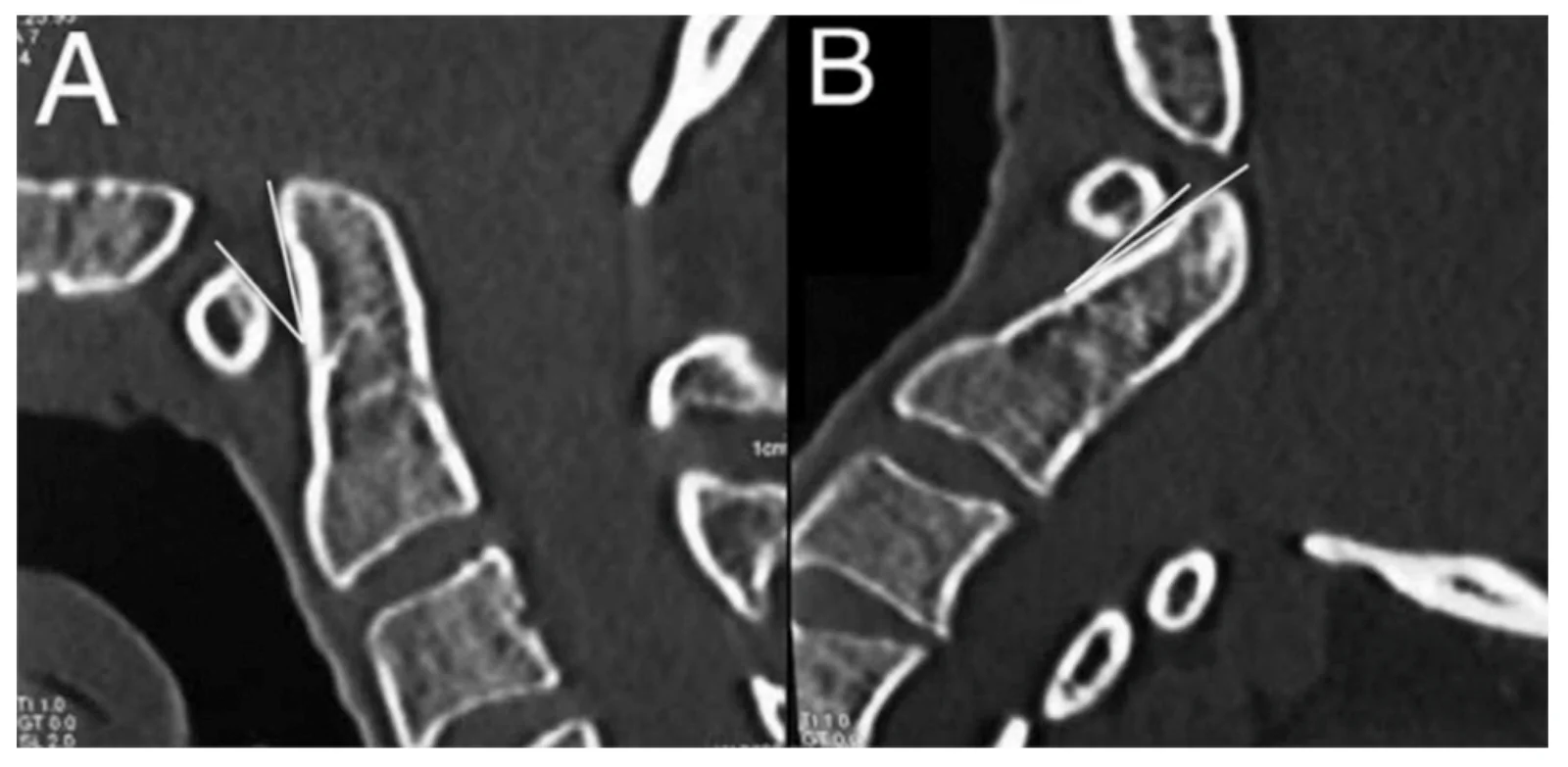
C1 anterior arch-odontoid angle. (**A**) Pre-op; (**B**) post-op.

In addition to these two measurement parameters, the angle between the posterior margin of the anterior arch of C1 and the anterior surface of the odontoid process was evaluated as our proposed third parameter (Figure 2).

### 2.4. Statistical Analysis

Data were analyzed using SPSS v29.0 software (IBM Corp., Armonk, NY, USA). Measurement reliability was assessed separately for preoperative and postoperative images. Interobserver reliability was determined by comparing the mean of the two measurements obtained by Observer 1 (N.K.) with the mean of the two measurements obtained by Observer 2 (E.E.). Intraobserver reliability was assessed separately for each observer by comparing their first and second measurements. Reliability was quantified using the intraclass correlation coefficient (ICC) with corresponding 95% confidence intervals (CIs). A two-way random-effects model with absolute agreement was used for ICC analysis, as both observers were considered representative of a larger population of potential observers and absolute agreement between measurements was of interest. For interobserver reliability, the mean of the two measurements obtained by each observer was compared. Because the intended clinical use of these parameters is a single measurement performed by a single observer, the single-measure ICC(2,1) was regarded as the primary interobserver estimate, whereas the average-measure ICC(2,2) is additionally reported to describe the reliability of the averaged measurements used in the comparative analyses; both were obtained from the same two-way random-effects model with absolute agreement. ICC values were interpreted according to commonly used reliability thresholds, with values ≥0.90 considered excellent, 0.75–0.89 considered good, 0.50–0.74 considered moderate, and <0.50 considered poor.

The distribution of continuous variables was assessed using the Shapiro–Wilk test. Preoperative and postoperative radiological measurements were compared using the paired-samples *t*-test for normally distributed variables and the Wilcoxon signed-rank test for non-normally distributed variables. Effect sizes were reported as Cohen’s d_z (mean paired difference divided by the SD of the paired differences) for paired-samples t-tests and as r = Z/√n for Wilcoxon signed-rank tests. For paired-samples t-tests, the mean paired difference (postoperative minus preoperative) and its 95% CI were reported, whereas for Wilcoxon signed-rank tests, the Hodges–Lehmann estimate of the median paired difference and its 95% CI were reported. Measurement error was assessed using the standard error of measurement (SEM) and smallest detectable change (SDC). Relative measurement error was evaluated using the coefficient of variation (CV), and agreement between repeated measurements was assessed using Bland–Altman analysis. Because 11 craniovertebral measurements were tested simultaneously, *p*-values were adjusted for multiple comparisons using the Benjamini–Hochberg procedure to control the false discovery rate (FDR) at 5%, implemented with the STATS PADJUST extension command in IBM SPSS Statistics 29.0. Both unadjusted *p*-values and FDR-adjusted q-values were reported, with q < 0.05 considered statistically significant for the 11 craniovertebral measurements. For separate prespecified comparisons, including comparisons between cranial and caudal AADI measurements, unadjusted *p*-values were used, with *p* < 0.05 considered statistically significant. To test whether the relationship between the cranial and caudal AADI changed after surgery, the within-patient difference score (Cranial AADI−Caudal AADI) was computed at each time point and its paired change was analyzed. All tests were two-sided, and continuous variables were presented as mean ± standard deviation (SD) and/or median (min–max), as appropriate.

## 3. Results

Between November 2000 and December 2022, 26 patients underwent craniovertebral junction stabilization. Of these, 18 were male (69%), and 8 were female (31%), with a mean age of 46 years (range, 20–74 years). The etiologies were traumatic in 11 patients (42%), congenital in 10 (38%), and neoplastic in 5 (19%) (Table 1). Screw–plate fixation was used in two cases, both in 2002, whereas screw–rod fixation was used in the remaining 24 cases. Before 2010, fixation frequently extended to the lower cervical levels, including C7 in one case. From 2011 onward, the lowest fixation level was C3 or above in all cases, with C2 being the most commonly selected lowest fixation level (Table 1).

The cohort comprised 11 patients with traumatic conditions (42.3%), 10 with congenital conditions (38.5%), and 5 with neoplastic conditions (19.2%). Two patients included in the traumatic group also had an underlying inflammatory disorder: one had ankylosing spondylitis, and one had rheumatoid arthritis. Screw–rod constructs were used in 24 patients (92.3%), whereas screw–plate constructs were used in 2 patients (7.7%). According to the caudal extent of stabilization, the lowest instrumented vertebral level was C2 in 10 patients (38.5%), C3 in 7 (26.9%), and C4-C7 in 9 (34.6%) (Table 1).

Measurement error and interobserver agreement were calculated separately for each parameter (Table 2). For the linear measurements, intraobserver SEM ranged from 0.28 to 2.71 mm and interobserver SEM from 0.87 to 3.37 mm. The smallest measurement error was observed for the cranial and caudal AADI: intraobserver SEM was 0.31–0.44 mm for Observer 1 and 0.55–0.90 mm for Observer 2, and interobserver SEM was 0.87–1.40 mm, corresponding to an SDC of 2.42–3.87 mm; the postoperative measurements were the most precise (interobserver SEM 0.94 mm for the cranial and 0.87 mm for the caudal AADI). The largest linear error was observed for the McGregor’s line–odontoid measurement (interobserver SEM 2.79 mm preoperatively and 3.37 mm postoperatively). For the angular measurements, intraobserver SEM ranged from 0.70° to 7.38° and interobserver SEM from 2.37° to 10.79°, with the clivodens (9.44° preoperatively and 10.79° postoperatively) and clivus canal angles (7.04° and 9.75°) showing the highest and the C0-C1 angle the lowest absolute errors (2.37° and 2.75°). Relative error was low for the clivus canal and clivodens angles (CV, 4.8–7.5%) and moderate for the AADI and the smaller angular parameters (CV, 12.5–31.8%), the latter reflecting their small mean values rather than large absolute errors; the CV was not calculated for the Chamberlain, McGregor’s and McRae’s line–odontoid measurements, whose mean values approach zero and render the coefficient uninterpretable.

Reliability and absolute error were interpreted separately, as a high ICC does not imply a small absolute error. Despite good-to-excellent ICCs, the interobserver SDC ranged from 6.50 to 9.34 mm for the line–odontoid distances and 7.61–29.90° for the angular parameters, so that only large individual changes exceed measurement error. The segmented AADI showed the smallest error of all linear parameters (intraobserver SDC 1.05 mm cranial and 1.18 mm caudal; interobserver SDC 3.30 mm and 2.61 mm). The mean postoperative reduction (−1.49 mm and −1.25 mm) marginally exceeded the intraobserver but not the interobserver SDC, and at the individual level the change exceeded the intraobserver SDC in 8 of 15 and 6 of 15 patients and the interobserver SDC in 3 and 2 patients, respectively.

Bland–Altman analysis showed negligible mean bias between observers across all parameters, with 95% CIs that included zero in every comparison (Table 2). For the newly proposed measurements, the interobserver bias was 0.00 mm for the cranial AADI (95% CI −1.09 to 1.09 preoperatively and −0.74 to 0.74 postoperatively) and 0.00 mm for the caudal AADI (95% CI −0.79 to 0.79 and −0.68 to 0.68). The 95% limits of agreement were −3.87 to 3.87 mm preoperatively (95% CI of the lower limit, −5.78 to −1.96; of the upper limit, 1.96 to 5.78) and −2.61 to 2.61 mm postoperatively (−3.90 to −1.32 and 1.32 to 3.90) for the cranial AADI, and −2.79 to 2.79 mm (−4.16 to −1.41 and 1.41 to 4.16) and −2.42 to 2.42 mm (−3.62 to −1.22 and 1.22 to 3.62) for the caudal AADI. These were the narrowest limits of agreement among all linear parameters, whereas the widest were observed for McGregor’s line–odontoid measurement (−9.35 to 9.34 mm postoperatively) among the linear and for the clivodens angle (−29.92 ° to 29.92° postoperatively) among the angular measurements. The absolute difference between observers was not related to the magnitude of the measurement for the cranial or caudal AADI preoperatively (r = 0.42, *p* = 0.123 and r = 0.49, *p* = 0.061), indicating no relevant proportional bias, although a weak association was present for the cranial AADI postoperatively (r = 0.56, *p* = 0.030).

Interobserver and intraobserver reliability were high across all radiological measurements. Intraobserver ICCs ranged from 0.897 to 0.983 preoperatively and from 0.880 to 0.987 postoperatively. Except for the McGregor’s line–odontoid measurement for Observer 2 (ICC = 0.897 preoperatively and 0.887 postoperatively) and the atlantoaxial angle for Observer 1 postoperatively (ICC = 0.880), all intraobserver ICCs were ≥0.90, indicating excellent reliability. For interobserver reliability, both single-measure and average-measure estimates are reported. Single-measure interobserver ICCs, which reflect the reliability of a single measurement obtained by a single observer, ranged from 0.784 to 0.899 preoperatively and from 0.791 to 0.880 postoperatively, indicating good reliability for all measurements; the lowest values were observed for the McGregor’s line–odontoid measurement (ICC = 0.784 preoperatively and 0.791 postoperatively) and the highest for the caudal AADI (ICC = 0.899 preoperatively) and the clivus canal angle (ICC = 0.892 preoperatively). Average-measure interobserver ICCs, which reflect the reliability of the mean of two measurements, were correspondingly higher (0.879–0.947 preoperatively and 0.904–0.939 postoperatively), being excellent for all measurements except the McGregor’s line–odontoid measurement (ICC = 0.879) and the C1 anterior arch-odontoid angle (ICC = 0.894) preoperatively, which showed good reliability. All ICC estimates were statistically significant (*p* < 0.001) (Table 3).

Due to inadequate image quality, incomplete visualization, postoperative metallic artifacts, or inability to reliably identify the relevant anatomical landmarks, not all radiological measurements could be obtained in every patient. Paired analyses were therefore based on 14–16 patients, depending on the parameter, as reported in Table 3. Because measurement availability may have been related to anatomical complexity, underlying pathology, imaging characteristics, or the fixation construct, the missing observations could not be assumed to be completely random. Odontoid tip distance measurements relative to the Chamberlain, McGregor, and McRae lines were obtained. The odontoid process was indicated as “+” if located above the line and “–“ if below it.

**Figure 3:**
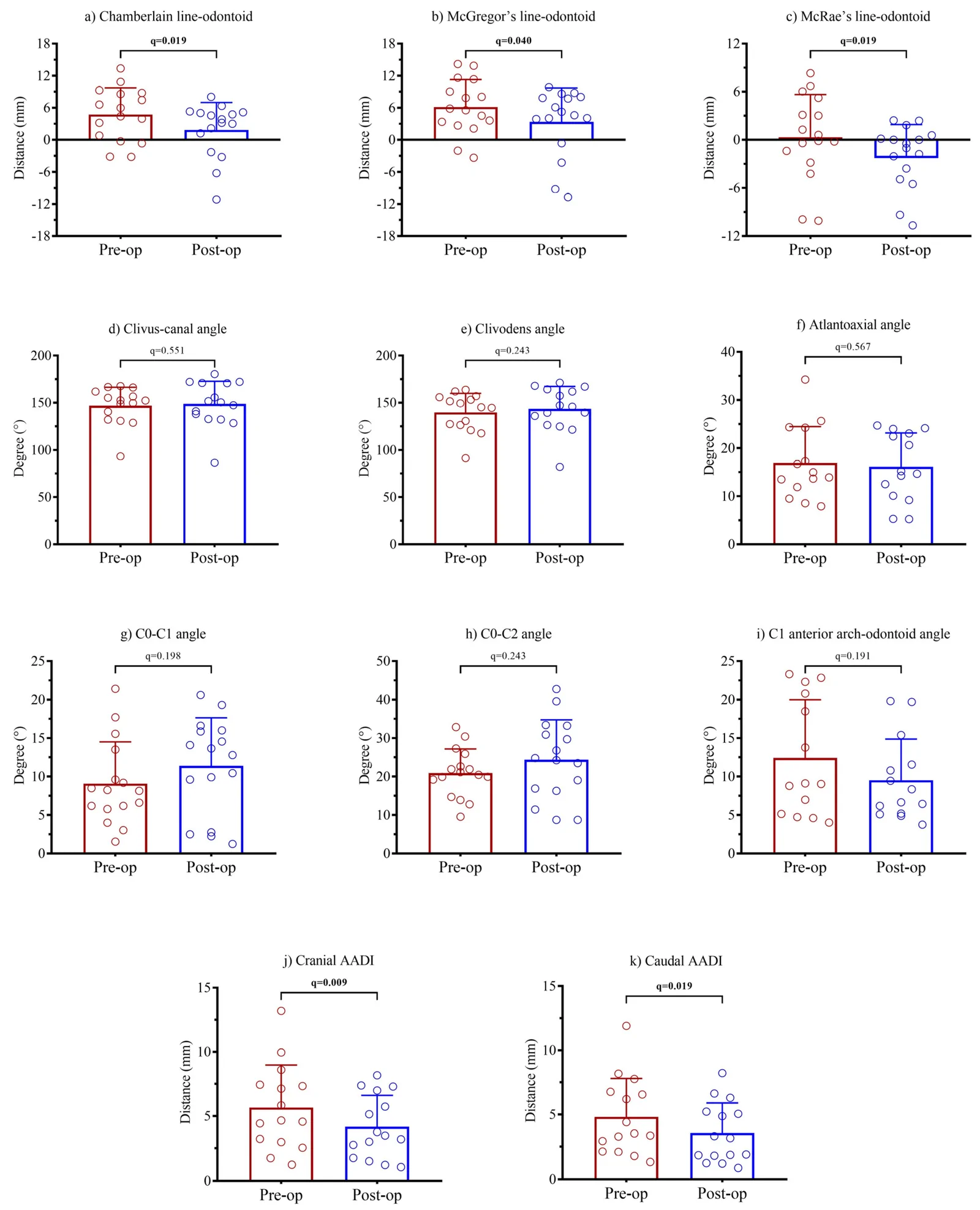
Distribution and comparison of preoperative and postoperative craniocervical junction measurements (**a**–**k**). Bars represent mean ± SD, and individual points indicate individual subjects. Paired t-test or Wilcoxon signed-rank test was used as appropriate. *p*-values were adjusted using the Benjamini–Hochberg procedure, and q < 0.05 was considered statistically significant.

The distributions of preoperative and postoperative measurements are shown in Figure 3, and the corresponding effect sizes and confidence intervals are presented in Table 3. Postoperative measurements showed significant reductions in the Chamberlain line–odontoid distance [4.74 ± 4.98 vs. 1.86 ± 5.11 mm; median, 5.2 (−3.2 to 13.4) vs. 3.5 (−11.2 to 8.0); r = −0.69; q = 0.019], McGregor’s line–odontoid distance [6.14 ± 5.16 vs. 3.37 ± 6,34 mm; median, 5.7 (−3.4 to 14.2) vs. 4.9 (−10.7 to 9.9); r = −0.58; q = 0.040], and McRae’s line–odontoid distance [0.32 ± 5.33 vs. −2.00 ± 3.90 mm; median, 0.2 (−10.1 to 8.3) vs. −0.7 (−10.7 to 2.4); Cohen’s d_z = −0.81; q = 0.019] (Table 4).

Both cranial and caudal AADI showed statistically significant reductions after surgery. Cranial AADI decreased from 5.66 ± 3.31 to 4.17 ± 2.44 mm [median, 4.7 (1.2 to 13.2) vs. 3.5 (1.1 to 8.2); r = −0.79; q = 0.009], while caudal AADI decreased from 4.81 ± 2.98 to 3.57 ± 2.32 mm [median, 3.5 (1.3 to 11.9) vs. 3.2 (0.9 to 8.2); r = −0.67; q = 0.019] (Table 4).

No statistically significant differences were observed after Benjamini–Hochberg adjustment for the clivus canal angle, clivodens angle, atlantoaxial angle, C0-C1 angle, C0-C2 angle, or C1 anterior arch-odontoid angle (all q > 0.05) (Table 3). Given the limited sample size, these non-significant findings should be interpreted with caution, as insufficient statistical power cannot be ruled out.

**Figure 4:**
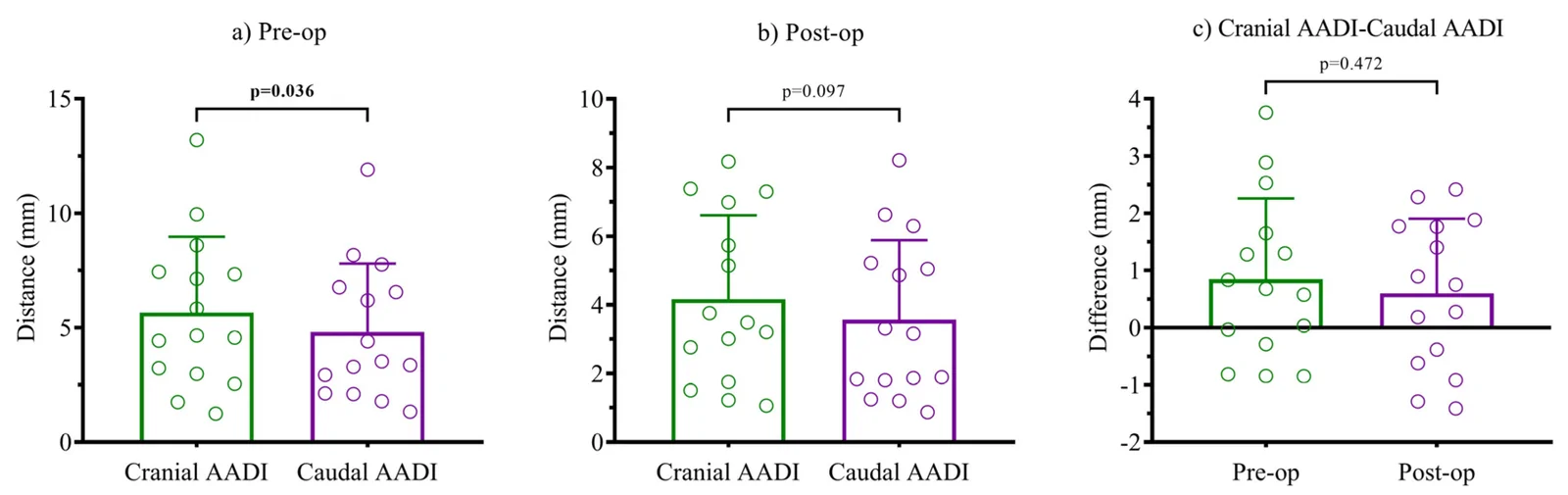
Cranial and caudal anterior atlantodental interval (AADI). (**a**) Paired preoperative cranial and caudal AADI values (paired *t*-test). (**b**) Paired postoperative values (paired *t*-test). (**c**) Within-patient difference score (Cranial AADI−Caudal AADI) preoperatively versus postoperatively (paired t-test). Bars represent mean ± SD, and individual points indicate individual subjects.

Preoperatively, the cranial AADI was significantly greater than the caudal AADI (5.66 ± 3.31 vs. 4.81 ± 2.98 mm; mean paired difference 0.85 mm, 95% CI 0.07 to 1.63; *p* = 0.036; Cohen’s d_z = 0.60) (Figure 4a), whereas postoperatively no statistically significant difference was observed between the cranial and caudal AADI (4.17 ± 2.44 vs. 3.57 ± 2.32 mm; mean paired difference 0.60 mm, 95% CI −0.12 to 1.32; *p* = 0.097; Cohen’s d_z = 0.46) (Figure 4b). Because a significant preoperative comparison and a non-significant postoperative comparison do not by themselves indicate a change in the cranial–caudal relationship, the within-patient difference score (cranial AADI—caudal AADI) was compared directly between time points; this difference score was 0.85 ± 1.41 mm preoperatively and 0.60 ± 1.31 mm postoperatively, and the paired change was not statistically significant (mean change −0.25 mm, 95% CI −0.96 to 0.47; *p* = 0.472; Cohen’s d_z = −0.19, 95% CI −0.75 to 0.36) (Figure 4c).

Preoperative neurological examination findings were available for 26 patients and demonstrated a heterogeneous neurological profile. Eleven patients (42.3%) had no documented motor or sensory neurological deficit at presentation, whereas the remaining patients had varying degrees of neurological impairment, including monoparesis, hemiparesis, quadriparesis, and paraplegia. Hyperreflexia, pathological reflexes, clonus, and sensory disturbances were documented in several patients. Severe neurological deficits, including sphincter dysfunction and loss of anal and/or cremasteric reflexes, were also present in some patients.

At discharge, postoperative neurological examinations were considered evaluable in 22 patients, while the remaining records were excluded because of death or insufficient, uncertain, or unavailable documentation. Among the evaluable patients, most had no newly documented neurological deficit, and patients with pre-existing neurological impairment generally remained neurologically stable or showed partial improvement in motor or sensory function. One patient developed right hemiplegia secondary to a postoperative middle cerebral artery infarction, representing a new neurological deficit. Another patient developed postoperative dysphagia without a new motor or sensory neurological deficit. Overall, no consistent pattern of postoperative neurological deterioration was observed among the evaluable patients.

## 4. Discussion

Patients with CVJ pathology may experience a wide range of severe clinical consequences due to neural compression and autonomic dysfunction, including blood pressure fluctuations, arrhythmias, respiratory disturbances, and even sudden death [3,15,16]. Therefore, accurate and reliable radiological assessment is essential for diagnosing these pathologies, guiding treatment decisions, and evaluating surgical outcomes.

Historically, several craniovertebral measurements have been used for evaluating CVJ anomalies, including the Chamberlain line, McGregor’s line, and McRae line [15,17]. The existence of numerous measurement techniques reflects the inherent difficulty of diagnosing CVJ anomalies, partly because of substantial anatomical variability in healthy populations. For example, in basilar invagination, one of the most widely accepted diagnostic criteria is an odontoid process extending more than 3 mm above the Chamberlain line, which is considered pathological. However, this criterion is known to have a considerable false-positive rate [18,19]. Similarly, the clivodens angle measured on sagittal CT reconstructions can vary considerably even among healthy individuals. Consequently, numerous angular measurements have been proposed, such as the clivus canal angle [20], cervicomedullary angle [21], foramen magnum angle [22], C0-C1 angle [23], C0-C2 angle [24], clivopalate angle [25] and clivodens angle [26], to improve the assessment of CVJ alignment and diagnostic precision.

In the present study, the odontoid tip distances from the Chamberlain, McGregor, and McRae lines showed significant postoperative changes following stabilization. These findings demonstrate responsiveness in this cohort but do not establish diagnostic accuracy or universal reliability. Traditional craniometric measurements are influenced by substantial anatomical variability, with overlapping reference values between healthy and pathological populations that may result in false-positive classifications (18,19). Their application also depends on accurate identification of landmarks such as the posterior hard palate, basion, and opisthion, which may be difficult in patients with congenital craniovertebral anomalies, incomplete imaging coverage, altered head position, or postoperative metallic artifacts. Although the clivus canal and clivodens angles tended to increase postoperatively, these changes did not reach statistical significance. Similarly, no significant postoperative changes were observed in the atlantoaxial, C0-C1, or C0-C2 angles. Therefore, no conclusion regarding the superiority of either traditional linear or angular measurements can be drawn from the present findings.

Craniovertebral kyphosis is increasingly recognized as an important component of deformity, particularly in patients with basilar invagination and atlantoaxial dislocation. Previous studies have demonstrated that surgical reduction and stabilization can restore craniovertebral alignment, although this correction has generally been evaluated using angular parameters such as the clivo-axial and C0-C2 angles [6,8,9]. In the present study, we explored whether the local relationship between the anterior arch of C1 and the odontoid process could provide a simpler representation of this deformity. Both cranial and caudal AADI showed significant reductions following stabilization. Although cranial AADI was significantly greater than caudal AADI preoperatively and the postoperative comparison was not statistically significant, direct within-patient analysis showed that the cranial–caudal difference score did not change significantly after stabilization (mean change, −0.25 mm; 95% CI, −0.96 to 0.47; *p* = 0.472; Cohen’s dz = −0.19). Therefore, the observed reductions do not demonstrate postoperative convergence or differential local realignment between the cranial and caudal measurement locations. Segmented AADI measurements reached statistical significance after FDR adjustment, whereas the evaluated angular parameters did not. However, because the magnitudes of change and standardized effect sizes were not directly compared, this difference in statistical significance should not be interpreted as evidence of greater responsiveness or sensitivity of segmented AADI. Conventional AADI primarily represents translational displacement between C1 and the dens and is closely related to transverse ligament integrity and upper cervical kinematics [12,14]. However, because it is recorded as a single distance, it does not describe variation along the cranial–caudal extent of the C1–dens interface. Segmented AADI may therefore be considered an extension of conventional AADI rather than an alternative. For the proposed cranial and caudal AADI measurements, the single-measure interobserver ICC(2,1), which is more relevant to the intended clinical use of a single measurement performed by one observer, indicated good reliability. The higher average-measure ICC(2,2) values reflected the reliability of the averaged measurements used in the comparative analyses and should not be interpreted as evidence of excellent reliability for a single clinical measurement. Moreover, ICC is a relative measure and does not necessarily indicate small absolute measurement error. Although segmented AADI had the smallest SDC among the evaluated linear parameters, the mean postoperative reductions in cranial and caudal AADI marginally exceeded the intraobserver SDC but remained below the interobserver SDC. At the individual level, the observed change exceeded the intraobserver SDC in 8 of 15 patients for cranial AADI and 6 of 15 patients for caudal AADI, whereas it exceeded the interobserver SDC in only 3 and 2 patients, respectively. Thus, the postoperative reductions observed at the group level should not be assumed to represent changes exceeding measurement error in every individual patient. Reproducibility alone also does not establish diagnostic or construct validity, and the present findings should not be interpreted as a formal validation of segmented AADI as a direct measure of craniovertebral kyphosis. The study did not include a healthy control group or an independent reference standard for craniovertebral kyphosis. Moreover, the C1 anterior arch-odontoid angle did not demonstrate a statistically significant postoperative change. Accordingly, the observed postoperative changes should be regarded as exploratory radiological findings and not as evidence of the diagnostic or clinical validity of segmented AADI.

The present cohort included traumatic, congenital, and neoplastic conditions and differed in terms of fixation devices and the caudal extent of stabilization. These conditions may differ substantially in bone morphology, reducibility, baseline craniovertebral alignment, and the intended degree of surgical correction. Moreover, occipitocervical fixation techniques evolved during the 22-year study period from plate-based and relatively long constructs to modern screw–rod systems and shorter constructs. Therefore, the underlying pathology, fixation strategy, lowest instrumented level, and surgical period may all have influenced the magnitude of postoperative radiological change.

Pooling these conditions was considered appropriate for the present exploratory analysis because the primary objective was to determine whether the evaluated measurements could detect within-patient changes following stabilization rather than to compare clinical or surgical outcomes among etiological groups. The paired design allowed each patient to serve as their own reference, thereby reducing, but not eliminating, the influence of interindividual anatomical variability. Subgroup and sensitivity analyses were considered; however, the etiological subgroups were small (11 traumatic, 10 congenital, and 5 neoplastic cases), the fixation-device groups were markedly imbalanced (24 screw–rod and 2 screw–plate constructs), and the fixation levels were distributed across several categories. These subgroup comparisons would therefore have been inadequately powered and potentially unstable. The pooled findings should consequently be interpreted as exploratory and should not be considered universally applicable across all craniovertebral junction pathologies or fixation strategies.

This study has several limitations. Its retrospective, single-center design, small sample size, and heterogeneous cohort, including traumatic, congenital, inflammatory, and neoplastic conditions, limit the generalizability of the findings. The 22-year study period also involved changes in imaging technology, surgical techniques, instrumentation, and stabilization strategies that may have influenced postoperative alignment. Although neutral positioning was intended for all CT examinations, exact equivalence of head and neck position between the preoperative and postoperative scans could not be verified retrospectively. Residual differences in flexion or extension may have influenced the AADI and angular measurements and may therefore represent a potential source of measurement bias. The small sample size limited statistical power, and the non-significant findings for some angular measurements should therefore be interpreted cautiously. Some measurements could not be obtained because of inadequate image quality, incomplete visualization, postoperative metallic artifacts, or difficulty identifying the required anatomical landmarks. These limitations may have been associated with anatomical complexity, underlying pathology, imaging period, or fixation construct; therefore, the missing data may have been systematic rather than completely random. Restricting the analyses to patients with assessable paired measurements may consequently have selected patients with more readily identifiable anatomy and introduced selection bias. The small number of available observations precluded a reliable formal analysis of missingness. Although observer reproducibility was assessed, the study did not establish the diagnostic or clinical validity, thresholds, or superiority of the proposed cranial and caudal AADI measurements. Standardized clinical and functional outcomes and revision surgery were also not systematically assessed; therefore, radiological changes cannot be assumed to represent clinically meaningful improvement.

Further prospective studies with larger, more homogeneous cohorts, standardized imaging and surgical protocols, healthy controls, and clinical–radiological correlation are needed. Normal reference ranges for cranial and caudal AADI should also be established in healthy populations before their diagnostic utility can be determined. Nevertheless, these measurements may have potential value for postoperative radiological follow-up.

## 5. Conclusions

Classical craniovertebral measurements, including the distances between the odontoid tip and the Chamberlain, McGregor, and McRae lines, demonstrated significant within-patient postoperative changes in this cohort; however, these findings should not be interpreted as evidence of diagnostic accuracy or universal reliability. Separate cranial and caudal AADI measurements also demonstrated significant postoperative changes. Segmented AADI and angular parameters showed different patterns of postoperative change, but their relative responsiveness was not directly compared. Accordingly, segmented AADI should currently be regarded as an exploratory radiological parameter rather than a clinically validated measure of craniovertebral kyphosis. Its diagnostic thresholds and association with clinical outcomes remain to be established in prospective studies with healthy controls, standardized imaging protocols, and clinical–radiological correlations.

## Figures and Tables

**Table 1:** Etiology and surgical technique of the included patients.

Year	Sex	Age	Etiology	Occipital Fixation Material	Cervical Fixation Material	Lowest Level	Cervical Vertebrae Fixation Technique
2000	M	72	Trauma	Screw–plate	Screw–rod	C3	C2 PIAS, C3 LM
2001	M	46	Trauma	Screw–plate	Screw–rod	C3	C1-C2 PTAS, C3 LM
2002	F	72	Trauma	Screw–plate	Screw–plate	C5	C3-4-5 LMS
2002	M	28	Congenital	Screw–plate	Screw–plate	C4	C3-4 LMS
2004	M	42	Trauma	Screw–plate	Screw–rod	C4	C3-4 LMS
2005	M	45	Tumor	Screw–plate	Screw–rod	C5	C3-4-5 LMS
2005	M	73	Tumor	Screw–plate	Screw–rod	C5	C3-4-5 LMS
2006	M	26	Congenital	Screw–plate	Screw–rod	C5	C3-4-5 LMS
2007	M	40	Trauma	Screw–plate	Screw–rod	C4	C3-4 LMS
2008	M	20	Trauma	Screw–plate	Screw–rod	C3	C2 PS, C3 LMS
2008	F	45	Trauma	Screw–plate	Screw–rod	C2	C2 ILS
2009	F	24	Tumor	Screw–plate	Screw–rod	C6	R:C4-5-6 LMS, L:C4 LMS, C5-6 TFS
2010	M	74	Trauma	Screw–plate	Screw–rod	C7	C2-C7 PS, C3-4-5-6 LMS
2011	M	51	Congenital	Clamp–screw–plate	Screw–rod	C3	C2 PS, R: C3 LMS, L: C3 PS
2011	F	42	Congenital	Screw–plate	Screw–rod	C3	C2-3 PS
2011	F	40	Congenital	Screw–plate	Screw–rod	C2	C2 PS
2012	M	42	Trauma	Screw–plate	Screw–rod	C2	C2 PS
2013	M	20	Congenital	Screw–plate	Screw–rod	C2	C2 PS
2015	F	59	Tumor	Screw–plate	Screw–rod	C2	C2 PS
2016	M	32	Congenital	Screw–plate	Screw–rod	C2	C2 ILS
2020	F	46	Congenital	Screw–plate	Screw–rod	C2	C2 ILS
2020	M	57	Tumor	Screw–plate	Screw–rod	C3	C2-3 PS
2021	M	54	AS + Trauma	Clamp–screw–plate	Screw–rod	C3	C2-3 PS
2021	M	74	RA + Trauma	Screw–plate	Screw–rod	C2	C2 PS
2021	F	47	Congenital	Screw–plate	Screw–rod	C2	C1 LMS, C2 SS
2022	M	49	Congenital	Screw–plate	Screw–rod	C2	C2 ILS

Abbreviations: PS: pedicle screw, PIAS: pars interarticularis screw, LMS: lateral mass screw, PTAS: posterior transarticular screw, ILS: intra laminar screw, SS: spinous screw, TFS: transfacet screw, R: right, L: left, AS: ankylosing spondylitis, RA: rheumatoid arthritis.

**Table 2:** Measurement error and interobserver agreement for each radiological parameter on pre-op and post-op CT images.

Measurement	Time Point	Intraobserver SEM (SDC) Observer 1/Observer 2	Interobserver SEM (SDC)	CV (%)	Interobserver Mean Bias (95% CI)	Lower Limit of Agreement (95% CI)	Upper Limit of Agreement (95% CI)
**Chamberlain line–odontoid**	Pre-op	1.39 (3.85)/1.54 (4.26)	2.41 (6.68)	NA	0.00 (−1.82 to 1.82)	−6.69 (−9.86 to −3.51)	6.69 (3.51 to 9.86)
**Chamberlain line–odontoid**	Post-op	1.47 (4.06)/1.66 (4.59)	2.35 (6.50)	NA	0.00 (−1.77 to 1.77)	−6.51 (−9.60 to −3.42)	6.51 (3.42 to 9.60)
**McGregor’s line–odontoid**	Pre-op	1.06 (2.94)/2.24 (6.21)	2.79 (7.74)	NA	0.00 (−2.11 to 2.11)	−7.74 (−11.42 to −4.07)	7.75 (4.07 to 11.42)
**McGregor’s line–odontoid**	Post-op	1.07 (2.97)/2.71 (7.51)	3.37 (9.34)	NA	0.00 (−2.54 to 2.54)	−9.35 (−13.78 to −4.91)	9.34 (4.91 to 13.78)
**McRae’s line–odontoid**	Pre-op	0.99 (2.73)/1.81 (5.02)	2.49 (6.90)	NA	0.00 (−1.88 to 1.88)	−6.90 (−10.18 to −3.63)	6.90 (3.63 to 10.18)
**McRae’s line–odontoid**	Post-op	0.71 (1.97)/1.37 (3.79)	1.76 (4.88)	NA	0.00 (−1.33 to 1.33)	−4.88 (−7.20 to −2.56)	4.88 (2.56 to 7.20)
**Clivus canal angle**	Pre-op	3.16 (8.75)/5.04 (13.95)	7.04 (19.51)	4.8	0.00 (−5.52 to 5.52)	−19.53 (−29.17 to −9.88)	19.53 (9.88 to 29.17)
**Clivus canal angle**	Post-op	4.48 (12.42)/5.73 (15.87)	9.75 (27.01)	6.6	0.00 (−7.64 to 7.64)	−27.03 (−40.38 to −13.67)	27.03 (13.67 to 40.38)
**Clivodens angle**	Pre-op	4.45 (12.32)/6.93 (19.18)	9.44 (26.16)	6.8	0.00 (−7.40 to 7.40)	−26.18 (−39.11 to −13.24)	26.18 (13.24 to 39.11)
**Clivodens angle**	Post-op	5.94 (16.45)/7.38 (20.44)	10.79 (29.90)	7.5	0.00 (−8.45 to 8.45)	−29.92 (−44.70 to −15.14)	29.92 (15.13 to 44.70)
**Atlantoaxial angle**	Pre-op	1.85 (5.12)/1.99 (5.51)	3.45 (9.55)	20.4	0.00 (−2.81 to 2.81)	−9.56 (−14.49 to −4.62)	9.56 (4.62 to 14.49)
**Atlantoaxial angle**	Post-op	2.56 (7.09)/2.04 (5.64)	2.82 (7.81)	17.5	0.00 (−2.30 to 2.30)	−7.82 (−11.86 to −3.79)	7.82 (3.78 to 11.85)
**C0-C1 angle**	Pre-op	1.30 (3.60)/1.79 (4.95)	2.37 (6.57)	26.1	0.00 (−1.79 to 1.79)	−6.57 (−9.69 to −3.45)	6.57 (3.45 to 9.69)
**C0-C1 angle**	Post-op	1.34 (3.72)/1.98 (5.50)	2.75 (7.61)	24.1	0.00 (−2.07 to 2.07)	−7.62 (−11.23 to −4.00)	7.62 (4.00 to 11.23)
**C0-C2 angle**	Pre-op	1.49 (4.14)/1.44 (3.99)	2.61 (7.23)	12.5	0.00 (−1.97 to 1.97)	−7.24 (−10.67 to −3.80)	7.24 (3.80 to 10.67)
**C0-C2 angle**	Post-op	2.39 (6.63)/2.47 (6.84)	4.06 (11.23)	16.6	0.00 (−3.06 to 3.06)	−11.24 (−16.58 to −5.90)	11.24 (5.90 to 16.58)
**C1 anterior arch-odontoid angle**	Pre-op	1.93 (5.34)/2.97 (8.22)	3.95 (10.95)	31.8	0.00 (−3.23 to 3.23)	−10.96 (−16.61 to −5.30)	10.96 (5.30 to 16.61)
**C1 anterior arch-odontoid angle**	Post-op	0.70 (1.94)/1.72 (4.77)	2.42 (6.71)	25.5	0.00 (−1.98 to 1.98)	−6.72 (−10.18 to −3.25)	6.72 (3.25 to 10.18)
**Cranial AADI**	Pre-op	0.44 (1.22)/0.90 (2.49)	1.40 (3.87)	24.7	0.00 (−1.09 to 1.09)	−3.87 (−5.78 to −1.96)	3.87 (1.96 to 5.78)
**Cranial AADI**	Post-op	0.31 (0.85)/0.55 (1.51)	0.94 (2.61)	22.6	0.00 (−0.74 to 0.74)	−2.61 (−3.90 to −1.32)	2.61 (1.32 to 3.90)
**Caudal AADI**	Pre-op	0.53 (1.48)/0.64 (1.76)	1.01 (2.79)	20.9	0.00 (−0.79 to 0.79)	−2.79 (−4.16 to −1.41)	2.79 (1.41 to 4.16)
**Caudal AADI**	Post-op	0.28 (0.77)/0.56 (1.55)	0.87 (2.42)	24.5	0.00 (−0.68 to 0.68)	−2.42 (−3.62 to −1.22)	2.42 (1.22 to 3.62)

SEM: standard error of measurement, SDC: smallest detectable change, CV: coefficient of variation, CI: confidence interval, AADI: Anterior Atlantodental Interval, NA: not applicable. Values are in mm for the linear and in degrees for the angular measurements. SEM was calculated as the standard deviation of the differences divided by √2 and SDC as 2.77 × SEM. Intraobserver values are based on the two repeated measurements of each observer, whereas interobserver values and the Bland–Altman estimates are based on the difference between the mean of the two measurements of Observer 1 and the mean of the two measurements of Observer 2. Limits of agreement were calculated as the mean bias ± 1.96 standard deviations of the differences. The CV was not calculated for the Chamberlain, McGregor’s and McRae’s line–odontoid measurements because their mean values approach zero, which renders the coefficient uninterpretable.

**Table 3:** Intraobserver and interobserver reliability of radiological measurements on preoperative and postoperative CT images, reported as single-measure and average-measure intraclass correlation coefficients (ICC).

Measurement	Observer 1 Pre-op ICC(2,1)	Observer 2 Pre-op ICC(2,1)	Interobserver Pre-op ICC(2,1)	Interobserver Pre-op ICC(2,2)	Observer 1 Post-op ICC(2,1)	Observer 2 Post-op ICC(2,1)	Interobserver Post-op ICC(2,1)	Interobserver Post-op ICC(2,2)
**Chamberlain line–odontoid**	0.936 (0.821–0.978)	0.947 (0.851–0.982)	0.820 (0.554–0.933)	0.905 (0.715–0.968)	0.925 (0.793–0.974)	0.943 (0.840–0.980)	0.835 (0.587–0.939)	0.919 (0.757–0.973)
**McGregor’s line–odontoid**	0.960 (0.888–0.986)	0.897 (0.721–0.964)	0.784 (0.480–0.919)	0.879 (0.635–0.960)	0.974 (0.924–0.991)	0.887 (0.696–0.961)	0.791 (0.493–0.922)	0.911 (0.737–0.970)
**McRae’s line–odontoid**	0.968 (0.909–0.989)	0.927 (0.797–0.975)	0.830 (0.576–0.937)	0.908 (0.722–0.969)	0.969 (0.911–0.989)	0.921 (0.781–0.973)	0.840 (0.598–0.941)	0.910 (0.730–0.970)
**Clivus canal angle**	0.965 (0.862–0.989)	0.952 (0.863–0.984)	0.892 (0.708–0.963)	0.943 (0.829–0.981)	0.954 (0.819–0.986)	0.961 (0.888–0.987)	0.867 (0.648–0.954)	0.929 (0.786–0.976)
**Clivodens angle**	0.948 (0.854–0.982)	0.926 (0.794–0.975)	0.830 (0.562–0.940)	0.907 (0.719–0.969)	0.941 (0.837–0.980)	0.938 (0.825–0.979)	0.838 (0.581–0.943)	0.912 (0.735–0.971)
**Atlantoaxial angle**	0.949 (0.855–0.983)	0.959 (0.882–0.986)	0.840 (0.569–0.946)	0.918 (0.753–0.973)	0.880 (0.682–0.958)	0.940 (0.832–0.980)	0.871 (0.643–0.957)	0.930 (0.791–0.977)
**C0-C1 angle**	0.945 (0.848–0.981)	0.937 (0.822–0.978)	0.848 (0.617–0.945)	0.933 (0.800–0.978)	0.943 (0.840–0.981)	0.916 (0.770–0.971)	0.847 (0.613–0.944)	0.911 (0.733–0.970)
**C0-C2 angle**	0.948 (0.851–0.983)	0.958 (0.873–0.986)	0.860 (0.644–0.949)	0.911 (0.718–0.971)	0.963 (0.892–0.988)	0.967 (0.901–0.989)	0.874 (0.676–0.954)	0.939 (0.809–0.980)
**C1 anterior arch-odontoid angle**	0.937 (0.821–0.979)	0.916 (0.764–0.972)	0.808 (0.497–0.935)	0.894 (0.664–0.966)	0.978 (0.919–0.993)	0.922 (0.776–0.974)	0.825 (0.534–0.941)	0.904 (0.696–0.969)
**Cranial AADI**	0.983 (0.951–0.994)	0.955 (0.873–0.985)	0.867 (0.648–0.954)	0.929 (0.786–0.976)	0.984 (0.956–0.995)	0.962 (0.892–0.987)	0.874 (0.665–0.956)	0.933 (0.798–0.978)
**Caudal AADI**	0.969 (0.912–0.989)	0.960 (0.889–0.986)	0.899 (0.726–0.965)	0.947 (0.841–0.982)	0.987 (0.960–0.995)	0.951 (0.864–0.983)	0.880 (0.677–0.958)	0.936 (0.808–0.979)

ICC: Intraclass correlation coefficient, AADI: anterior atlantodental interval. Values are ICCs with 95% CIs in parentheses, all from a two-way random-effects model with absolute agreement. Intraobserver reliability compared the two measurements of each observer using the single-measure ICC(2,1); interobserver reliability compared the mean of each observer’s two measurements and is given both as the single-measure ICC(2,1), reflecting one measurement by one observer, and the average-measure ICC(2,2), reflecting averaged measurements. ICCs were interpreted as excellent (≥0.90), good (0.75–0.89), moderate (0.50–0.74) or poor (<0.50). All estimates were significant (*p* < 0.001).

**Table 4:** Comparison of craniovertebral junction radiological measurements before and after surgery.

Measurement	n	Pre-op Mean ± SD	Post-op Mean ± SD	* Mean Change (95% CI)	Effect Size	*p*	*q (BH)*
Chamberlain line–odontoid (mm)	16	4.74 ± 4.98	1.86 ± 5.11	−3.09 (−4.74 to −0.77)	r = −0.69	**0.003**	**0.019**
McGregor’s line–odontoid (mm)	16	6.14 ± 5.16	3.37 ± 6.34	−3.11 (−5.34 to −0.63)	r = −0.58	**0.018**	**0.040**
McRae’s line–odontoid (mm)	16	0.32 ± 5.33	−2.00 ± 3.90	−2.32 (−3.85 to −0.79)	d_z = −0.81	**0.006**	**0.019**
Clivus canal angle (°)	15	146.90 ± 19.57	148.74 ± 24.10	1.84 (−3.85 to 7.53)	d_z = 0.18	0.501	0.551
Clivodens angle (°)	15	139.83 ± 20.15	143.53 ± 23.73	3.70 (−2.18 to 9.58)	d_z = 0.35	0.199	0.243
Atlantoaxial angle (°)	14	16.88 ± 7.61	16.10 ± 7.06	−0.78 (−3.64 to 2.08)	d_z = −0.16	0.567	0.567
C0-C1 angle (°)	16	9.08 ± 5.43	11.38 ± 6.25	2.30 (−0.74 to 5.34)	d_z = 0.41	0.126	0.198
C0-C2 angle (°)	16	20.91 ± 6.28	24.38 ± 10.36	3.47 (−1.91 to 8.85)	d_z = 0.34	0.189	0.243
C1 anterior arch-odontoid angle (°)	14	12.42 ± 7.55	9.52 ± 5.35	−2.14 (−5.61 to 0.25)	r = −0.44	0.104	0.191
Cranial AADI (mm)	15	5.66 ± 3.31	4.17 ± 2.44	−1.24 (−2.53 to −0.35)	r = −0.79	**<0.001**	**0.009**
Caudal AADI (mm)	15	4.81 ± 2.98	3.57 ± 2.32	−1.02 (−2.07 to −0.30)	r = −0.67	**0.007**	**0.019**

AADI: Anterior Atlantodental interval, SD: standard deviation. * The 95% CI represents the mean of the paired differences (post-op minus pre-op) for the paired t-test and the Hodges–Lehmann median of the paired differences for the Wilcoxon tests. Effect sizes are reported as Cohen’s d_z for paired-samples t-tests and r = Z/√n for Wilcoxon signed-rank tests. *p*-values were adjusted for multiple testing using the Benjamini–Hochberg false discovery rate (FDR); both unadjusted *p*-values and adjusted q-values are reported, with q < 0.05 considered significant. Sample sizes vary because some landmarks were not assessable on all images.

## Data Availability

The data presented in this study are available from the corresponding author upon reasonable request. The data are not publicly available due to patient privacy and ethical restrictions.

## Abbreviations

The following abbreviations are used in this manuscript:

AADI	Anterior atlantodental interval
BH	Benjamini–Hochberg
CI	Confidence interval
CT	Computed tomography
CV	Coefficient of variation
CVJ	Craniovertebral junction
FDR	False discovery rate
ICC	Intraclass correlation coefficient
SD	Standard deviation
SDC	Smallest detectable change
SEM	Standard error of measurement
SPSS	Statistical Package for the Social Sciences
